# Nationwide Outcome after Pancreatoduodenectomy in Patients at Very High Risk (ISGPS-D) for Postoperative Pancreatic Fistula

**DOI:** 10.1097/SLA.0000000000006174

**Published:** 2023-12-11

**Authors:** Rutger T. Theijse, Thomas F. Stoop, Tessa E. Hendriks, J. Annelie Suurmeijer, F. Jasmijn Smits, Bert A. Bonsing, Daan J. Lips, Eric Manusama, Erwin van der Harst, Gijs A. Patijn, Jan H. Wijsman, Mark Meerdink, Marcel den Dulk, Ronald van Dam, Martijn W.J. Stommel, Kees van Laarhoven, Roeland F. de Wilde, Sebastiaan Festen, Werner A. Draaisma, Koop Bosscha, Casper H.J. van Eijck, Olivier R. Busch, I. Quintus Molenaar, Bas Groot Koerkamp, Hjalmar C. van Santvoort, Marc G. Besselink

**Affiliations:** * Department of Surgery, Amsterdam UMC, location University of Amsterdam, Amsterdam, the Netherlands; † Cancer Center Amsterdam, Amsterdam, the Netherlands; ‡ Department of Surgery, Division of Surgical Oncology, University of Colorado, Anschutz Medical Campus, Aurora, CO; § Department of Surgery, Leiden University Medical Center, Leiden, the Netherlands; ∥ Department of Surgery, Regional Academic Cancer Center Utrecht, University Medical Center Utrecht and St. Antonius Hospital Nieuwegein, Utrecht University, the Netherlands; ¶ Department of Surgery, Medisch Spectrum Twente, Enschede, the Netherlands; # Department of Surgery, Medisch Centrum Leeuwarden, the Netherlands; ** Department of Surgery, Maasstad Hospital, Rotterdam, the Netherlands; †† Department of Surgery, Isala Clinics, Zwolle, the Netherlands; ‡‡ Department of Surgery, Amphia Hospital, Breda, the Netherlands; §§ Department of Surgery, University Medical Center Groningen, Groningen, the Netherlands; ∥∥ Department of Surgery, Maastricht University Medical Center, Maastricht, the Netherlands; ¶¶ Department of General, Visceral and Transplant Surgery, University Hospital Aachen, Germany; ## NUTRIM-School of Nutrition and Translational Research in Metabolism, Maastricht University, Maastricht, the Netherlands; *** Department of Surgery, Radboud University Medical Center, Nijmegen, the Netherlands; ††† Department of Surgery, Erasmus MC Cancer Institute, University Medical Center, Rotterdam, the Netherlands; ‡‡‡ Department of Surgery, OLVG Oost, Amsterdam, the Netherlands; §§§ Department of Surgery, Jeroen Bosch Hospital, ‘s Hertogenbosch, the Netherlands

**Keywords:** Pancreatoduodenectomy, mortality, POPF, ISGPS-D, total pancreatectomy

## Abstract

**Objective:**

To assess nationwide surgical outcome after pancreatoduodenectomy (PD) in patients at very high risk for postoperative pancreatic fistula (POPF), labeled as International Study Group for Pancreatic Surgery (ISGPS) category D.

**Background:**

Morbidity and mortality after ISGPS-D PD is perceived so high that a recent randomized trial advocated prophylactic total pancreatectomy (TP) as alternative aiming to lower this risk. However, current outcomes of ISGPS-D PD remain unknown as large nationwide series are lacking.

**Methods:**

Nationwide retrospective analysis including consecutive patients undergoing ISGPS-D PD (ie, soft texture and pancreatic duct diameter ≤3 mm), using the mandatory Dutch Pancreatic Cancer Audit (2014-2021). Primary outcome was in-hospital mortality, and secondary outcomes included major morbidity (ie, Clavien-Dindo grade ≥IIIa) and POPF (ISGPS grade B/C). The use of prophylactic TP to avoid POPF during the study period was assessed.

**Results:**

Overall, 1402 patients were included. In-hospital mortality was 4.1% (n=57), which decreased to 3.7% (n=20/536) in the last 2 years. Major morbidity occurred in 642 patients (45.9%) and POPF in 410 (30.0%), which corresponded with failure-to-rescue in 8.9% (*n*=57/642). Patients with POPF had increased rates of major morbidity (88.0% vs. 28.3%; *P*<0.001) and mortality (6.3% vs. 3.5%; *P*=0.016) compared to patients without POPF. Among 190 patients undergoing TP, prophylactic TP to prevent POPF was performed in 4 (2.1%).

**Conclusions:**

This nationwide series found a 4.1% in-hospital mortality after ISGPS-D PD with 45.9% major morbidity, leaving little room for improvement through prophylactic TP. Nevertheless, given the outcomes in the 30% of patients who develop POPF, future randomized trials should aim to prevent and mitigate POPF in this high-risk category.

Postoperative pancreatic fistula (POPF) remains the most important cause of surgery-related morbidity and mortality after pancreatoduodenectomy (PD).^1^ Although POPF can mostly be managed with antibiotics and minimally invasive drainage when early recognized,^2^ those requiring any type of invasive intervention remain associated with postoperative mortality up to 18%.^3,4^ Risk prediction of POPF can guide and optimize perioperative management. Therefore, the International Study Group for Pancreatic Surgery (ISGPS) recently developed a simple classification system, distinguishing patients undergoing PD into 4 risk categories (A–D) for developing POPF based on main pancreatic duct diameter and pancreatic texture. The very high-risk category D is defined by presence of both soft texture of the pancreatic parenchyma and a main pancreatic duct diameter ≤3 mm.^5^

Prophylactic total pancreatectomy (TP) has been suggested as an alternative to PD in patients at very high risk for POPF (ie, categorized as ISGPS-D), aiming to avoid severe POPF-related morbidity and mortality.^6–11^ Recently, a randomized trial reported the short-term benefits of such an approach^7^ but was criticized for not including mortality and long-term quality of life as the main end points.^12^ Postoperative outcome following TP has improved in recent years, particularly in high-volume centers.^13–16^ Nonetheless, the use and outcome following TP varies widely among countries,^13,17^ and the resulting lifelong endocrine and exocrine insufficiency remain formidable.^16,18,19^

The Dutch nationwide PORSCH trial demonstrated that early recognition and step-up management of POPF improved failure-to-rescue (from 15% to 8%) and reduced the rates of both morbidity and mortality after pancreatic surgery on a national level.^2^ These improved outcomes question the need for prophylactic TP in patients undergoing ISGPS-D PD, particularly since this “high-risk” group comprises approximately one-third of the patients who undergo a PD.^5^ The in-hospital mortality of prophylactic TP varies from 3.3% to 7.0%,^7,8^ whereas the current mortality after ISGPS-D PD is unknown as nationwide multicenter data are lacking. Hence, to accurately assess the extent of this clinical dilemma, it is of paramount importance to investigate current outcomes after ISGPS-D PD before considering any prophylactic measures.

Therefore, this nationwide multicenter study aimed to assess the rates of in-hospital mortality, major morbidity, and POPF after PD in patients categorized as ISGPS-D, and the nationwide use of prophylactic TP to avoid POPF.

## METHODS

This observational retrospective study was performed in accordance with the Strengthening the Reporting of Observational Studies in Epidemiology guidelines.^20^ Data were obtained through the mandatory Dutch Pancreatic Cancer Audit (DPCA),^21^ as part of the Dutch Pancreatic Cancer Group, comprising all centers performing pancreatic surgery in the Netherlands.^22^ The study protocol was approved by the scientific committee of the Dutch Pancreatic Cancer Group.^22^

### Study Design and Patients

This nationwide analysis included all consecutive patients who underwent open or minimally invasive PD at very high risk for POPF classified in the ISGPS-D risk category (ie, both pancreatic duct ≤3 mm and soft pancreatic parenchyma) from January 1^st^, 2014 until December 31^st^, 2021. Texture of the pancreatic parenchyma was subjectively assessed and reported by the operating surgeon, whereas the diameter of the main pancreatic duct was measured on preoperative imaging. Patients undergoing PD with missing data regarding either pancreatic parenchyma texture or pancreatic duct size were excluded. Patients who developed a POPF grade B/C were compared with patients who did not (ie, no POPF/biochemical leak). Furthermore, patients who underwent primary elective TP (in any ISGPS risk category) during the study period were identified from the DPCA. Indications of TP (eg, if prophylactic TP was performed to prevent POPF) were additionally collected from the preoperative multidisciplinary team conference and the operation reports. Notably, it was not our intention to directly compare these patient groups (ie, ISGPS-D PD and prophylactic TP for the prevention of POPF).

### End points

The primary end point was mortality, defined as in-hospital mortality. Secondary end points included in-hospital/30-day major morbidity (ie, Clavien-Dindo grade ≥IIIa^23^), the rate of POPF grade B/C, and the use of prophylactic TP performed to prevent POPF.

### Definitions

Histopathological diagnoses were defined in accordance with the World Health Organization definitions.^24,25^ The preoperative physical condition of patients was classified according to the American Society of Anaesthesiologists-Physical Score (ASA-PS). In addition to the ISGPS classification system, the updated alternative Fistula Risk Score (ua-FRS), was calculated for each patient.^26^

PD was defined following the ISGPS definition and comprised the pylorus-preserving pancreatoduodenectomy, pylorus resecting pancreatoduodenectomy, and “classic” Whipple procedure. The extent of PD was defined as follows: extended resection(s) comprised (sub)total gastrectomy, (hemi)colectomy, and vascular resections. Vascular resections comprised resection of the portomesenteric venous axis, superior mesenteric artery, hepatic artery, and/or the celiac axis. TP was defined as either preoperatively planned (ie, elective) or intraoperatively converted from partial pancreatectomy to TP.^27^ Type of portomesenteric vein resections was classified in accordance with the ISGPS definition and categorized into types 1 to 4.^28^

All pancreatic surgery-specific complications [ie, POPF, delayed gastric emptying, postpancreatectomy hemorrhage (PPH), bile leakage, and chyle leakage] were defined by the ISGPS and International Study Group of Liver Surgery.^29–33^ POPF was defined as grade B/C. For the other pancreatic surgery-specific complications, only grade B/C was considered to be of clinical relevance and included. Complications were registered when they occurred either during hospital stay or within 30 days after index surgery. The failure-to-rescue rate was calculated as mortality (numerator) among all patients with major morbidity (ie, Clavien-Dindo Grade ≥IIIa; denominator). Readmission was defined as admission to the hospital for any reason within 30 days following discharge, whereby patients who died within the hospital were excluded from calculations regarding the readmission rate.

### Statistical Analysis

Data analyses were performed with RStudio (software version 1.3.1093, Boston, MA).^34^ Two-tailed *P*-value of <0.050 was considered statistically significant. Descriptive statistics were used to summarize patient characteristics. Categorical variables were assessed using Pearson χ^2^ test, and Fisher exact test when appropriate, and are presented as frequencies and percentages. The Mann-Whitney *U* test was used to compare numerical variables, presented as medians with corresponding interquartile ranges.

First, a logistic regression analysis was performed within the PD group to determine potential predictors for mortality. The selected potential prognostic subgroups for mortality were included based on literature and expert opinion and comprised age, body mass index, ASA-PS, ua-FRS, extended resection(s), and postoperative pathology. To dichotomize patients based on ua-FRS, an optimal cutoff value for predicted probability was determined using the Youden index retrieved from the area under the ROC curve, whereby the balance between sensitivity and specificity was maximized. This cutoff value was subsequently tested as independent variable in logistic regression analysis. Results of the regression analyses are presented in odds ratios (OR) with corresponding 95% confidence intervals (CI). Clinical predictors with a *P* value <0.200 in univariable analysis were included in the multivariable analysis.^35^ Backward stepwise selection was used for the removal of insignificant variables in multivariable analysis until all remaining variables were statistically significant.

Subgroup analyses were performed whereby each subgroup associated with mortality, according to multivariable logistic regression, was separately assessed. Patients with and without POPF were compared to investigate its impact in each subgroup, respectively. Outcomes in grade C POPF were described separately. Of note, data regarding grade C POPF were only available in the period 2017-2021 (after the publication of the ISGPS fistula definition update in 2016).^29^ To evaluate the impact of the PORSCH approach, which was implemented in the Netherlands from January 2018 until November 2019,^2^ trend analysis using the Cochran-Armitage Test was performed assessing in-hospital mortality over time, comparing the time periods before, during (2018-2019), and after the PORSCH trial.

## RESULTS

### Baseline Characteristics

In total, 5808 patients underwent PD for all indications in all ISGPS risk categories, of whom 1886 patients (32.5%) were excluded due to missing pancreatic duct diameter (n=1618) and/or pancreatic parenchyma texture (n=581). Of the remaining 3992 patients, 1402 patients (35.7%) underwent PD categorized as ISGPS-D and were subsequently included in the study cohort.

Among these, PD was performed in 338 patients for pancreatic adenocarcinoma (24.3%) and in 627 patients for other nonpancreatic periampullary cancers (45.1%). Preoperative therapy was used in 113 patients (8.3%), of whom 84 patients (9.1%) received chemotherapy only and 24 patients (2.6%) chemoradiotherapy. In total, 190 patients (13.6%) underwent extended resection(s), of whom the majority underwent portomesenteric venous resection (n=113, 8.1%). All patient and treatment characteristics are summarized in Table 1.

**TABLE 1:** Baseline Characteristics ISGPS-D Pancreatoduodenectomy

Baseline characteristics*	ISGPS-D PD (n=1402)
Patient characteristics	
Sex, female, n (%)	631 (45.1)
Age, y, median [IQR]	68 [61–74]
ECOG PS≥2, n (%)	80 (5.8)
BMI, kg/m^2^, median [IQR]	25.3 (22.8–28.1)
ASA-PS≥2, n (%)	397 (28.5)
Treatment characteristics	
Preoperative therapy, n (%)	113 (8.3)
Chemotherapy	84 (9.1)
Chemoradiotherapy	24 (2.6)
Radiotherapy	5 (0.2)
Pancreatoduodenectomy, n (%)	—
Pylorus preserving	676 (48.2)
Pylorus resecting	177 (12.6)
Classic Whipple	549 (39.2)
Surgical approach, n (%)	–
Open	1.033 (74.6)
Robot-assisted	281 (20.3)
Laparoscopic	71 (5.1)
Pancreatic anastomosis, n (%)	—
Pancreatico-jejunostomy	1.255 (90.4)
Pancreatico-gastrostomy	52 (3.7)
Other/unknown	82 (5.9)
Colectomy, n (%)	48 (3.4)
(Sub)total gastrectomy, n (%)	21 (1.5)
Portomesenteric venous resection, n (%)	113 (8.1)
Type 1–2	71 (5.1)
Type 3–4	42 (3.0)
Arterial resection, n (%)	19 (1.4)
Pancreatic duct, mm, n (%)†	—
0–1	278 (19.8)
2	612 (43.7)
3	512 (36.5)
Operation time, minutes, median [IQR]	309 [246–378]
Blood loss, mL, median [IQR]	400 [200–756]
Pathology	
Diagnosis, n (%)	—
Pancreatic adenocarcinoma	338 (24.3)
Periampullary adenocarcinoma (nonpancreatic)	627 (45.1)
pNET	98 (7.0)
Benign/pre-malignant lesions†	194 (13.9)
Chronic pancreatitis	22 (1.6)
Other	112 (8.1)

All patients had a soft pancreas and a main pancreatic duct <3 mm.

* missing data: sex (n=2), BMI (n=25), ECOG (n=14), ASA-PS (n=7), preoperative therapy (n=38), surgical approach (n=17), pancreatico-enterostomosis (n=13), operation time (n=748), blood loss (n=690), and diagnosis (n=11).

† Intraductal papillary mucinous neoplasm, mucinous cystic neoplasm, solid pseudopapillary neoplasm, and adenoma.

ASA-PS indicates American Society of Anesthesiology Performance Score; BMI, body mass index; ECOG PS, Eastern Cooperative Oncology Group performance status; IQR, interquartile range; n, number of patients.

### Outcome ISGPS-D Pancreatoduodenectomy

The mortality rate of patients after ISGPS-D PD was 4.1% (n=57/1402). A major complication occurred in 45.9% (n=642/1402), which translates into a failure-to-rescue rate of 8.9% (57/642). Reoperation was necessary for 153 patients (10.9%) and 129 patients (9.2%) developed organ failure. In total, 418 patients (30.0%) developed POPF (grade B/C) and 166 patients (11.9%) PPH. All postoperative outcomes are summarized in Table 2.

**TABLE 2:** Postoperative Outcome in Patients With and Without POPF

		POPF		
Outcome measures*	Total (n=1402)	Yes (n=418)	No (*n*=975)	*P*
POPF (grade B/C), n (%)	418 (30.0)	—	—	—
PPH (grade B/C), n (%)	166 (11.9)	93 (22.4)	71 (7.3)	**<0.001**
Bile leakage (grade B/C), n (%)	128 (9.2)	63 (15.3)	64 (6.6)	**<0.001**
DGE (grade B/C), n (%)	366 (26.3)	186 (44.9)	178 (18.3)	**<0.001**
Major morbidity, n (%)	642 (45.9)	364 (87.1)	275 (28.2)	**<0.001**
Reoperation	153 (10.9)	37 (8.9)	19 (1.9)	**<0.001**
Organ failure	128 (9.1)	75 (17.9)	52 (5.3)	**<0.001**
MCU/ICU admission	193 (13.8)	106 (25.4)	86 (8.8)	**<0.001**
Mortality, n (%)	57 (4.1)	27 (6.5)	30 (3.1)	**0.004**
Failure-to-rescue, %	8.9	7.4	10.9	—
Postoperative hospital stay, days, median [IQR]	14 [9–24]	24 [15–39]	11 [8–17]	**<0.001**
Readmission, *n* (%)	296 (22.0)	129 (33.0)	168 (17.8)	**<0.001**

Percentages may not add up due to rounding and missing data.

* Missing data: POPF (n=9), chyle leakage (n=310), PPH (n=11), bile leakage (n=17), DGE (n=12), hospital stay (n=18), and readmission (n=7).

DGE indicates delayed gastric emptying; ICU, intensive care unit; IQR, interquartile range; MCU, medium care unit; n, number of patients; POPF, postoperative pancreatic fistula; PPH, postpancreatectomy hemorrhage.

### Impact of POPF

Patients who developed POPF had higher rates of pancreatic surgery-specific complications as compared with patients without POPF: PPH (22.4% vs. 7.3%; *P*<0.001), bile leakage (15.3% vs. 6.6%; *P*<0.001), and delayed gastric emptying (44.9% vs. 18.3%; *P*<0.001). In addition, in these patients the rate of major morbidity was increased over three-fold (87.1% vs. 28.2%; *P*<0.001), comprising higher rates of reoperation (8.9% vs. 1.9%; *P*<0.001), organ failure (17.9% vs. 5.3%; *P*<0.001), and mortality (6.5% vs. 3.1%; *P*=0.004). Grade C POPF was observed in 35 patients (2.5%) in whom the mortality rate was 37.1% (n=13/35), as summarized Table 3.

**TABLE 3:** Subgroup Analysis in Patients With Grade C POPF

Outcome measures*	Grade C POPF (*n*=35)
PPH (grade B/C), n (%)	17 (48.6)
Major morbidity, n (%)	35 (100)
Reoperation	7 (20.0)
Organ failure	23 (65.7)
MCU/ICU admission	28 (80.0)
Mortality, n (%)	13 (37.1)
Failure-to-rescue, %	38.2
Hospital stay, days, median [IQR]	36 [14–72]
Readmission, n (%)	7 (31.8)

* Missing data: hospital stay (n=2), readmission (n=1).

ICU indicates intensive care unit; MCU, medium care unit; POPF, postoperative pancreatic fistula; PPH, postpancreatectomy hemorrhage.

### Predictors for Mortality and Time Trends

In multivariable logistic regression analysis, age ≥70 years [OR=3.241 (95% CI: 1.799–6.102)], ASA-PS >2 [OR=1.780 (95% CI: 1.009–3.116)], ua-FRS [OR=2.413 (95% CI: 1.362–4.218)], and extended resection(s) [OR=2.202 (95% CI: 1.074–4.226)] were identified as independent prognostic factors for in-hospital mortality. See Table 4 for the complete logistic regression analysis. In subgroup analyses, each predictor for mortality was separately evaluated, as summarized in Supplementary Tables 1–4, Supplemental Digital Content 1, http://links.lww.com/SLA/E960.

**TABLE 4:** Logistic Regression Analysis for Predictors of In-hospital Mortality

	Univariable analysis		Multivariable analysis	
Variables	OR (95% CI)	*P*	OR (95% CI)	*P*
Age, years				
<70	1 [reference]	—	1 [reference]	—
≥70	3.450 (1.954–6.385)	**<0.001**	3.241 (1.799–6.102)	**<0.001**
BMI, kg/m^2^				
18.5−25.0	1 [reference]	—	—	—
<18.5	1.133 (0.062–5.817)	0.905	—	—
>25.0	2.122 (1.196–3.941)	**0.013**	—	—
ASA-PS				
≤2	1 [reference]	—	1 [reference]	—
>2	2.099 (1.208–3.581)	**0.008**	1.780 (1.009–3.116)	**0.044**
ua-FRS				
≤56.13%	1 [reference]	—	1 [reference]	—
>56.13%	2.169 (1.242–3.730)	**0.005**	2.413 (1.362–4.218)	**0.002**
Surgical approach				
Open	1 [reference]	—	—	—
Robot-assisted	1.560 (0.458–4.029)	0.410	—	—
Laparoscopic	1.479 (0.778–2.675)	0.210	—	—
Extended resection(s)				
No	1 [reference]	—	1 [reference]	—
Yes	1.744 (0.876–3.257)	0.097	2.202 (1.074–4.226)	**0.023**
Pathology				
Pancreatic cancer	1 [reference]	—	—	—
Benign/pre-malign	1.401 (0.582–3.304 .)	0.440	—	—
Periampullary cancer (nonpancreatic)	1.277 (0.672−2.598)	0.470	—	—

ASA-PS indicates American Society of Anaesthesiologists Performance Score; BMI, body mass index; CI, confidence interval; OR, odds ratio; ua-FRS, updated alternative Fistula Risk Score.

Mortality rates for patients with POPF in the subgroups of patients with ASA-PS >2 (10.7% vs. 4.2%*, P*=0.012) and patients who underwent extended resection(s) (11.3% vs. 3.9%, *P*=0.062) were at least doubled compared to those within these subgroups who did not develop POPF.

Trend analyses for mortality, aiming to assess the impact of the PORSCH trial, found a non-significant decrease in mortality rates ranging from 4.5% (n=20/447), 4.1% (n=17/402), to 3.7% (n=20/536) (*P*=0.552), prior, during, and after the trial, respectively.

### Total Pancreatectomy

During the study period, 190 patients underwent TP, mainly for pancreatic adenocarcinoma (n=98, 51.6%). Overall, 79 patients underwent preoperatively planned, elective TP (41.6%), whereas the decision to convert from partial pancreatectomy to TP intraoperatively was made in 106 patients (55.8%). In the remaining 5 patients (2.6%), the reason to perform TP was unknown. In 4/190 patients (2.1%), the reason to perform TP instead of partial pancreatectomy was to avoid a high-risk pancreatic anastomosis. Other indications were to achieve radicality (n=61/190, 32.1%), technical issues (ie, vascular resection and/or reconstruction or extensive bleeding) (n=26/190, 13.7%), and other reasons (n=15/190, 7.9%).

## DISCUSSION

This first nationwide multicenter cohort study on the surgical outcome in patients undergoing PD categorized as ISGPS-D found a 4.1% rate of in-hospital mortality and 45.9% major morbidity, which translates into a failure-to-rescue rate of 8.9%. In the 30% of patients after ISGPS-D PD who developed POPF, the rates of major morbidity (88.0% vs. 28.3%; *P*<0.001) and mortality (6.3% vs. 3.1%; *P*=0.004) were considerably increased, as compared to patients without POPF. In the Netherlands, during the 8-year study period, in only 4 of 190 patients (2.1%), TP was performed prophylactically to avoid POPF.

Studies reporting on surgical outcomes in patients undergoing PD in the ISGPS-D risk category are scarce. The 30.0% rate of POPF in the current study is somewhat higher than the 23.2% reported in the original ISGPS risk classification^5^ and also higher than the ≤19% benchmark for PD as established in 23 international high-volume centers.^36^ The 4.1% mortality after ISGPS-D PD in the current study is nearly 3 times higher than the benchmark (≤1.6%),^36^ although mortality decreased to 3.7% in the period following the PORSCH trial. Separate analysis revealed that patients with poor ASA-PS and those undergoing extended resection(s) were at higher risk for failure-to-rescue from POPF with increased mortality rates. Furthermore, the high rates of major morbidity (88%) and mortality (6.3%) in patients with POPF after ISGPS-D PD demonstrates that despite the excellent failure-to-rescue rate, focus should remain on preventing POPF.

A single-center study assessing 3000 consecutive PDs revealed that the rates of POPF and its clinical burden have remained stable over the past 20 years (ranging from 20.1% to 24.5%),^37^ whereas data from the North American (NSQIP) and Dutch (DPCA) registries even reported increasing rates of POPF after PD.^38,39^ These results further emphasize that prevention and mitigation of POPF following PD (even in high-volume expert centers) must be seen as an unmet clinical need, particularly in patients within the ISGPS-D risk category. Numerous strategies to prevent/mitigate (the severity of) POPF have been proposed, mainly focusing on the surgical technique of pancreatic anastomosis,^40^ somatostatin analogues (eg, pasireotide),^41^ hydrocortisone,^42^ intraoperative coverage of the hepatic artery and gastroduodenal artery stump,^43^ and the value of pancreatic ductal stenting,^44,45^ but have not gained general acceptance.^46^ Moreover, despite the ability to stratify patients by POPF risk, it remains challenging to predict patients at risk for the most severe fistulas (grade C POPF) and its associated high mortality of 37.1%.^47^ Subsequently, robust treatment strategies for patients in high-risk categories remain to be debated.^46,48,49^ This is underlined by the similarly high mortality rate in patients with grade C POPF found in the current study (37.1%).

This prompts the question whether prophylactic TP to avoid POPF should be used in patients undergoing ISGPS-D PD. Do the short-term benefits of prophylactic TP overrule the lifelong absence of endocrine and exocrine pancreatic function? The recently published PAN-IT trial, randomly assigned patients categorized as ISGPS-D between PD and TP with islet auto-transplantation (TP-IAT).^7^ Since the primary outcome (90-day overall morbidity) was higher following PD [OR=4.54 (95% CI: 1.07–19.3)] the authors concluded that TP-IAT may become the standard treatment in patients undergoing PD with high-risk intraoperative conditions (ie, ISGPS-D risk category). Of note, in-hospital mortality was non-significantly higher after PD [9.7% (n=3) vs. 3.3% (n=1); *P*=0.520], although the study was not powered to detect differences in 90-day mortality. However, it remains questionable whether reduction of postoperative (major) morbidity without improvement of postoperative mortality justifies a lifelong pancreatic state.^12^ In addition, ISGPS-D PD comprises a rather large group of 36% of all patients who underwent PD during the study period, which was associated with a 30% risk of grade B/C POPF. In general, most surgeons would consider a prophylactic TP in all these patients as too radical, although current literature strictly classifies “high-risk” PD as either ISGPS-D or an alternative fistula risk score >20% (ie, a chance of POPF occurrence of 20%). Therefore, this study also investigated potential subgroups of patients with ISGPS-D PD who might be at higher risk for mortality. This demonstrated that patients with poor performance status and patients undergoing extended resections are relevant prognostic subgroups for mortality, in whom nearly 3-fold increased mortality was seen.

Particularly, grade C POPF should be prevented, as international high-volume centers report a range of 0 to 12% POPF grade C.^36^ The rate of grade C POPF was 2.5% in the current study. These patients often have organ failure, which requires surgical intervention and sometimes even rescue pancreatectomy,^50^ associated with mortality rates up to 56%.^48^ If such patients could reliably be identified preoperatively, prophylactic TP may be considered as it is associated with lower mortality rates and complete absence of residual pancreatic function in both scenarios.^51^ Unfortunately, it remains highly challenging to predict POPF grade C and its associated outcomes and subsequently determine when/if minimally invasive interventions and/or pancreas-preserving procedures can be sufficient.^52^ As such, future research should focus on mortality and patient-reported outcomes. The currently recruiting TETRIS trial (NCT05212350) randomizes patients at high risk for POPF intraoperatively to TP or PD, potentially providing these insights.

The recent Dutch nationwide PORSCH trial showed that mortality following PD can be reduced by nearly 50% by implementing an algorithm for postoperative management.^2^ In line with these results, mortality rates in patients with ISGPS-D PD during the study period decreased gradually over time. The observed 3.7% mortality rate in the post-PORSCH period leaves little room for improvement with prophylactic TP(-IAT), which is therefore not considered of clinical relevance in patients requiring ISGPS-D PD in the Netherlands. This is reflected by the very low rate of prophylactic TP (1 procedure per 2 years) in the current study.

A recent systematic review and meta-analysis investigating the role of TP as an alternative to PD in 711 patients (334 TP and 373 PD) at high risk for POPF found a pooled rate of POPF of 40% following PD, which is higher than the 30% rate found in the current study.^53^ The meta-analysis showed similar 90-day mortality rates [6.3% vs. 6.2%; RR=1.04 (95% CI: 0.56–1.93)], whereas major morbidity was in favor of TP [26.7% *vs.* 38.3%; RR=0.65 (95% CI: 0.48–0.89)].^53^ However, this significant difference in major morbidity disappeared when analyzing only the matched/randomized studies (RR=0.73, 95% CI: 0.48–1.10). Strikingly, the pooled mortality following TP (6.8%) in the matched/randomized series is approximately 50% higher than the 4.1% mortality rate in the current study.

The results of the present study should be interpreted in the light of several limitations. First, the retrospective nature of this study introduces a risk of information bias, indicated by the exclusion of patients due to missing data. Second, due to the low number of TP performed as an alternative to PD in patients at risk of POPF, no direct comparison between both treatment groups was possible. Third, no data regarding overall survival was available, which may have been of particular interest in patients with pancreatic adenocarcinoma who developed POPF. Fourth, no data with regard to quality of life were available in the DPCA. Fifth, it is generally accepted that in complex surgery, 90-day postoperative outcome measures (ie, mortality and morbidity) best reflect the impact of surgery. Unfortunately, the DPCA only registers outcomes during in-hospital stay and up to 30 days after index surgery, for which the 90-day mortality and morbidity rates could not be reported. Sixth, no data are available on how duct size was determined on preoperative imaging (ie, computed tomography, MRI) or intraoperatively. The strength of the current study is its large sample size with nationwide data on the outcome of ISGPS-D PD.

## CONCLUSION

This nationwide series found an acceptable 4.1% in-hospital mortality after ISGPS-D PD, which decreased to 3.7% in the last 2 years of the study period. The likelihood that patients undergoing ISGPS-D would benefit from prophylactic TP seems very small. However, the mortality after ISGPS-D PD was more than doubled in patients with either unfavorable preoperative performance status or concomitant extended resection(s). Future randomized trials remain needed in patients with ISGPS-D PD to prevent POPF (or reduce its impact), with special attention for patients with additional risk factors.

## Supplementary Material
