# Supplementary material for: Nationwide Outcome after Pancreatoduodenectomy in Patients at Very High Risk (ISGPS-D) for Postoperative Pancreatic Fistula
Source: Ann Surg. 2023 Dec 11;281(2):322–8. doi: 10.1097/SLA.0000000000006174 (PMC11723487; doi:10.1097/SLA.0000000000006174)
Supplement: Supplementary file 1 [file sla-281-322-s001.docx]

| **SUPPLEMENTARY TABLE 2. Subgroup analysis in patients with ua-FRS ≥ 56.13%** | | | |
| --- | --- | --- | --- |
| **Outcome measures*** | **No** **POPF**  (*n*=209) | **POPF**  (*n*=135) | ***P* value** |
| PPH (grade B/C)**,** *n (%)* | 18 (8.7) | 45 (33.3) | **<0.001** |
| Major morbidity, *n (%)* | 70 (33.5) | 116 (85.9) | **<0.001** |
| Reoperation | 5 (2.4) | 11 (8.1) | **0.013** |
| Organ failure | 18 (8.6) | 34 (25.2) | **<0.001** |
| MCU / ICU admission | 24 (11.5) | 50 (37.0) | **<0.001** |
| Mortality**,** *n (%)* | 11 (5.3) | 12 (8.9) | 0.193 |
| Failure-to-rescue, % | 15.7 | 10.3 | - |
| Hospital stay, days, median [IQR] | 11 [8 – 19] | 30 [17 – 50] | **<0.001** |
| Readmission, *n* (%) | 34 (17.1) | 33 (26.8) | 0.056 |
| *POPF* postoperative pancreatic fistula; *PPH* postpancreatectomy hemorrhage; *MCU* medium care unit; *ICU* intensive care unit  *, Missing data: PPH (*n*=1), hospital stay (*n*=7), readmission (*n*=3) | | | |

| **SUPPLEMENTARY TABLE 1. Subgroup analysis in patients with age ≥ 70** | | | |
| --- | --- | --- | --- |
| **Outcome measures*** | **No POPF** (*n*=406) | **POPF**  (*n*=206) | ***P* value** |
| PPH (grade B/C)**,** *n (%)* | 28 (6.9) | 45 (22) | **<0.001** |
| Major morbidity, *n (%)* | 125 (30.8) | 186 (90.3) | **<0.001** |
| Reoperation | 32 (2.7) | 20 (9.7) | **<0.001** |
| Organ failure | 31 (7.6) | 41 (19.9) | **<0.001** |
| MCU / ICU admission | 44 (10.8) | 54 (26.2) | **<0.001** |
| Mortality**,** *n (%)* | 24 (5.9) | 17 (8.3) | 0.277 |
| Failure-to-rescue, % | 19.2 | 9.1 | - |
| Hospital stay, days, median [IQR] | 12 [9 – 19] | 26 [16 – 43] | **<0.001** |
| Readmission, *n* (%) | 66 (17.2) | 63 (33.3) | **<0.001** |
| *POPF* postoperative pancreatic fistula; *PPH* postpancreatectomy hemorrhage; *MCU* medium care unit; *ICU* intensive care unit  *, Missing data: PPH (*n*=3), hospital stay (*n*=9), readmission (*n*=3) | | | |

| **SUPPLEMENTARY TABLE 3. Subgroup analysis in patients with ASA-PS > 2** | | | |
| --- | --- | --- | --- |
| **Outcome measures*** | **No POPF** (*n*=265) | **POPF** (*n*=131) | ***P* value** |
| PPH (grade B/C)**,** *n (%)* | 26 (9.8) | 32 (24.6) | **<0.001** |
| Major morbidity, *n (%)* | 85 (32.1) | 119 (90.8) | **<0.001** |
| Reoperation | 9 (3.4) | 11 (8.4) | **0.033** |
| Organ failure | 20 (7.5) | 26 (19.8) | **<0.001** |
| MCU / ICU admission | 36 (13.6) | 38 (29.0) | **<0.001** |
| Mortality**,** *n (%)* | 11 (4.2) | 14 (10.7) | **0.012** |
| Failure-to-rescue, % | 12.9 | 11.8 | - |
| Hospital stay, days, median [IQR] | 12 [8 – 20] | 25 [16 – 39] | **<0.001** |
| Readmission, *n* (%) | 37 (14.6) | 40 (34.1) | **<0.001** |
| *ASA-PS* American Society of Anaesthesiologists Performance Score; *POPF* postoperative pancreatic fistula; *PPH* postpancreatectomy hemorrhage; *MCU* medium care unit; *ICU* intensive care unit  *, Missing data: PPH (*n*=2), hospital stay (*n*=7), readmission (*n*=1) | | | |

| **SUPPLEMENTARY TABLE 4. Subgroup analysis in patients with extended resection(s)** | | | |
| --- | --- | --- | --- |
| **Outcome measures*** | **No POPF** (*n*=127) | **POPF** (*n*=62) | ***P* value** |
| PPH (grade B/C)**,** *n (%)* | 11 (8.7) | 17 (27.4) | **<0.001** |
| Major morbidity, *n (%)* | 64 (50.4) | 57 (91.9) | **<0.001** |
| Reoperation | 2 (1.6) | 5 (8.1) | **0.039** |
| Organ failure | 8 (6.3) | 18 (29.0) | **<0.001** |
| MCU / ICU admission | 19 (15.0) | 22 (35.5) | **<0.001** |
| Mortality**,** *n (%)* | 5 (3.9) | 7 (11.3) | 0.062 |
| Failure-to-rescue, % | 7.8 | 12.3 | - |
| Hospital stay, days, median [IQR] | 14 [10 – 22] | 27 [16 – 43] | **<0.001** |
| Readmission, *n* (%) | 29 (23.8) | 14 (25.5) | 0.947 |
| *POPF* postoperative pancreatic fistula; *PPH* postpancreatectomy hemorrhage; *MCU* medium care unit; *ICU* intensive care unit  *, Missing data: PPH (*n*=1), hospital stay (*n*=2), readmission (*n*=1) | | | |
